# Virome in adult *Aedes albopictus* captured during different seasons in Guangzhou City, China

**DOI:** 10.1186/s13071-021-04922-z

**Published:** 2021-08-18

**Authors:** Wenqiao He, Yanxia Chen, Xiru Zhang, Mingji Peng, Da Xu, Huan He, Yuhan Gao, Junlin Chen, Jiarui Zhang, Zhiyu Li, Qing Chen

**Affiliations:** Department of Epidemiology, School of Public Health, Guangdong Provincial Key Laboratory of Tropical Disease Research, Southern Medical University, 1838 Guangzhou North Road, Guangzhou, 510515 China

**Keywords:** *Aedes albopictus*, Viral metagenomics, Season, Guangzhou

## Abstract

**Background:**

The mosquito *Aedes albopictus* is an important vector for many pathogens. Understanding the virome in *Ae. albopictus* is critical for assessing the risk of disease transmission, implementation of vector control measures, and health system strengthening.

**Methods:**

In this study, viral metagenomic and PCR methods were used to reveal the virome in adult *Ae. albopictus* captured in different areas and during different seasons in Guangzhou, China.

**Results:**

The viral composition of adult *Ae. albopictus* varied mainly between seasons. Over 50 viral families were found, which were specific to vertebrates, invertebrates, plants, fungi, bacteria, and protozoa. In rural areas, *Siphoviridae* (6.5%) was the most common viral family harbored by mosquitoes captured during winter and spring, while *Luteoviridae* (1.1%) was the most common viral family harbored by mosquitoes captured during summer and autumn. *Myoviridae* (7.0% and 1.3%) was the most common viral family in mosquitoes captured in urban areas during all seasons. Hepatitis B virus (HBV) was detected by PCR in a female mosquito pool. The first near full-length HBV genome from *Ae. albopictus* was amplified, which showed a high level of similarity with human HBV genotype B sequences. Human parechovirus (HPeV) was detected in male and female mosquito pools, and the sequences were clustered with HPeV 1 and 3 sequences.

**Conclusions:**

Large numbers of viral species were found in adult *Ae. albopictus*, including viruses from vertebrates, insects, and plants. The viral composition in *Ae*. *albopictus* mainly varied between seasons. Herein, we are the first to report the detection of HPeV and HBV in mosquitoes. This study not only provides valuable information for the control and prevention of mosquito-borne diseases, but it also demonstrates the feasibility of xenosurveillance.

**Graphical Abstract:**

**Figure:**
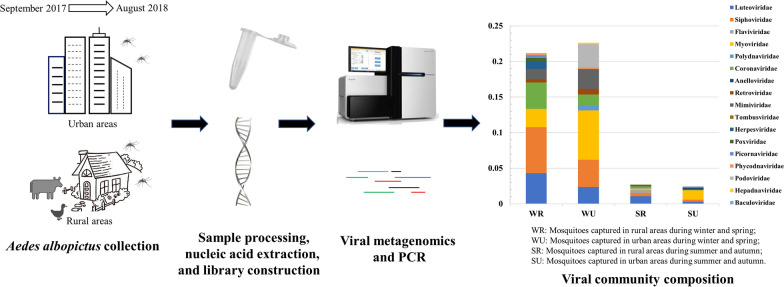

**Supplementary Information:**

The online version contains supplementary material available at 10.1186/s13071-021-04922-z.

## Background

Vector-borne infectious diseases affect over one billion people every year, leading to more than a million deaths globally [1]. *Aedes albopictus* (*Ae. albopictus*), a diurnal mosquito, is considered to be one of the most invasive animal species in the world. It was originally indigenous to the tropical and subtropical regions of southern Asia [2]. However, with the movement of humans and climate change, it has spread to many places in the world [3]. Except for Antarctica, *Ae. albopictus* is now found on all continents [4].

*Ae. albopictus* is a competent vector of at least 22 arboviruses that can cause human infections, including Zika virus (ZIKV), dengue virus (DENV), chikungunya virus (CHIKV), and yellow fever virus (YFV) [5]. Outbreaks caused by arboviruses have been reported, such as dengue fever in Thailand, Vietnam, and China, and chikungunya in France, Tanzania, Africa, and the Americas [2, 6–13]. ZIKV infections have also been reported in many countries, including Brazil, Nicaragua, and America [14–16].

China is a country located in East Asia. Most of the regions in this country are in the temperate zone, while some southern regions are located in the subtropical and tropical zones [17]. *Ae. albopictus* is found in nearly one third of the regions in China: south to Hainan Island, north to Shenyang and Dalian, west to Jingshui and Longnan, southwest to the Tibet Autonomous Region, and most regions to the east [18–20].

Some mosquito-borne diseases are prevalent in China. Since the first outbreak of dengue fever in 1978, this disease has been a threat for over 40 years in China, especially in southern and southeastern China [21]. *Ae. albopictus* was the sole vector responsible for some dengue fever outbreaks [22–26]. Outbreaks of chikungunya related to *Ae. albopictus* have also been reported in China [23, 27, 28]. Major public health concerns have been raised in China due to these frequent outbreaks [29].

Guangzhou (23°08′N, 113°16′E), the capital city of Guangdong Province, is located in southern China, and it is the fourth most populous city in the entire country [30]. The average annual temperature in Guangzhou is 22–23 °C, and the average rainfall is 1983 mm [31, 32]. *Ae. albopictus* is active in Guangzhou nearly all year round [33]. In 2014, there were 38,036 dengue cases reported in Guangzhou, accounting for 80.8% of all cases in the largest dengue outbreak in mainland China since 1990 [32, 34]. In addition, some sporadic cases of imported CHIKV infection were also reported in Guangzhou [35]. There is a great need for mosquito surveillance and control in Guangzhou.

Next-generation sequencing enables efficient detection of known and unknown viruses [36–38]. Viruses in *Aedes*, *Culex*, *Anopheles*, and *Armigeres* mosquitoes have been revealed using these methods. Some known viruses have been detected, such as DENV, ZIKV, and West Nile virus [39–43]. Novel viruses such as the Cuacua virus in *Mansonia* mosquitoes and the novel orbivirus in *Culex fatigans* mosquitoes have also been detected [37, 44]. Understanding the viral composition in mosquitoes is important for the prevention and control of emerging and reemerging mosquito-borne diseases. However, few studies have used next-generation sequencing methods to investigate the virome in *Ae. albopictus* [45, 46].

In this study, viral metagenomic and polymerase chain reaction (PCR) methods were used to reveal the viral composition in adult *Ae. albopictus* captured in different areas and during different seasons in Guangzhou City, China.

## Methods

### Sample collection

Between September 2017 and August 2018, adult *Ae. albopictus* specimens were collected three times per month in rural and urban areas in Guangzhou. The rural areas included Xiongwei and Nanfang villages in the Baiyun district, and the urban areas consisted of the Keyuan community in the Yuexiu district and the Taozhuang community in the Tianhe district. The captured mosquitoes were morphologically identified to determine their species and sex [37]. A total of 3346 adult *Ae. albopictus* were trapped (Additional file 1: Tables S1 and S2). All samples were stored at −80 °C in tubes containing RNAlater prior to processing.

### Laboratory viral metagenomic experiments

According to the collection sites and seasons, mosquitoes were randomly selected and pooled into four samples for viral metagenomic analysis (Additional file 1: Table S1). Pooled samples were homogenized in liquid nitrogen and then suspended in phosphate-buffered saline (PBS) [47, 48]. The supernatant was filtered through a 0.22-mm filter, and the filtered samples were then concentrated using centrifugal ultrafiltration tubes. To remove non-particle-protected nucleic acids, samples were incubated at 37 °C for 2 h with a mixture of DNases and RNase (New England Biolabs, USA). The total DNA and RNA of the pooled specimens was extracted using the MiniBEST Viral RNA/DNA Extraction Kit (TaKaRa, Japan). Reverse transcription was performed using the Transcriptor First Strand cDNA Synthesis Kit (Roche, Switzerland), and the primer used was reported in a previous study [49]. Random PCR was performed using the primer described in a previous study, and the purified PCR products were then obtained using the QIAquick Gel Extraction Kit (Qiagen, Germany) [49]. Libraries were constructed with the TruSeq™ DNA Sample Prep Kit (Illumina). The libraries were sequenced using the Illumina HiSeq platform at Shanghai Majorbio Bio-Pharm Technology Co., Ltd. (Shanghai, China) with 300-bp paired-end reads.

### Bioinformatics analysis of viral metagenomics

The quality score cut-off value was 20, and sequences with ambiguous bases (more than 10 bp N) and short length reads (less than 50 bp) were removed using the Sickle program (https://github.com/najoshi/sickle). To remove host-related sequences, quality reads were aligned with the host genome via BWA [50]. Reads with a high degree of similarity to the hosts’ genome were removed in further analyses. The taxonomic assignments were based on the National Center for Biotechnology Information (NCBI) nucleotide (NT) and non-redundant protein sequence (NR) databases, and the functional categories were based on the MetaGene system. In addition, the short reads were assembled using the IDBA-UD algorithm based on the de Bruijn graph approach [51]. The assembled contigs were analyzed based on the NCBI NR database. Phylogenetic analyses were performed using the MEGA 6.0 program with the maximum likelihood method.

### Extraction of nucleic acid and detection of viruses using PCR

Mosquitoes with the same sampling season, location, and sex were pooled into 196 samples (3–18 mosquitoes per sample) to perform PCR confirmatory tests (Additional file 1: Table S2). Using a MiniBEST Viral RNA/DNA Extraction Kit (TaKaRa, Japan), total RNA and DNA from these mosquito pools was extracted. Three arboviruses (ZIKV, DENV, and CHIKV) and some vertebrate viruses with high relative abundance in viral metagenomics were detected using PCR, including human parechovirus (HPeV), torque teno virus (TTV), coronavirus, herpesvirus, and hepatitis B virus (HBV) [52–58].

## Results

### Sample collection

A total of 990 mosquitoes were randomly selected for viral metagenomic analysis, and others (2356 *Ae. albopictus*) were pooled and applied to survey the prevalence and genomic diversity of the viruses using PCR (Additional file 1: Tables S1 and S2).

### Data overview of viral metagenomics

**Fig. 1:**
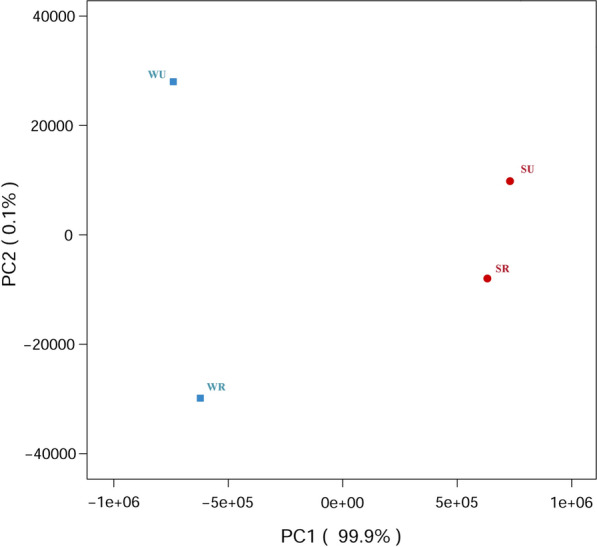
Principal component analysis (PCA) plots between mosquitoes captured at different locations and during different seasons. The viral composition mainly varied between the seasons. SR: mosquitoes captured in rural areas during summer and autumn; WR: mosquitoes captured in rural areas during winter and spring; SU: mosquitoes captured in urban areas during summer and autumn; WU: mosquitoes captured in urban areas during winter and spring

**Fig. 2:**
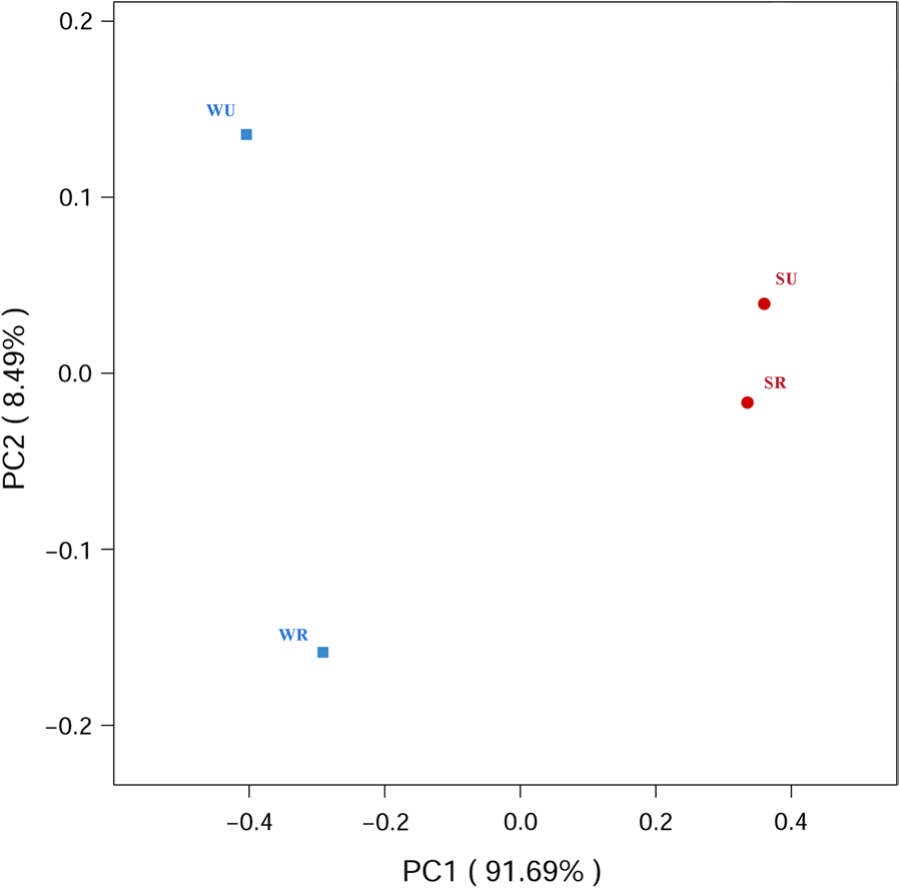
Principal coordinates analysis (PCoA) plots between mosquitoes captured at different locations and during different seasons. The viral composition mainly varied between the seasons. SR: mosquitoes captured in rural areas during summer and autumn; WR: mosquitoes captured in rural areas during winter and spring; SU: mosquitoes captured in urban areas during summer and autumn; WU: mosquitoes captured in urban areas during winter and spring

An average of 45,012,644 raw reads, 27,464,028 clean reads, 47,002 contigs, and 22,280 open reading frames (ORFs) was obtained for each sample (NCBI SRA number: SRP304029) (Additional file 1: Table S3). The majority of the sequences (over 80%) detected in our study were unidentifiable based on sequence similarity. Larger numbers of viral species were detected in mosquitoes captured during winter and spring as compared to mosquitoes captured during summer and autumn (Table 1). The viral composition in the mosquitoes mainly varied between seasons (Figs. 1 and 2).

**Table 1:** Number of families, genera and species of viruses detected in mosquitoes according to season and area

Mosquito group^a^	No. of families	No. of genera	No. of species
SR	40	65	245
SU	37	65	236
WR	42	74	254
WU	37	71	368

^a^SR: mosquitoes captured in rural areas during summer and autumn; WR: mosquitoes captured in rural areas during winter and spring; SU: mosquitoes captured in urban areas during summer and autumn; WU: mosquitoes captured in urban areas during winter and spring

**Fig. 3:**
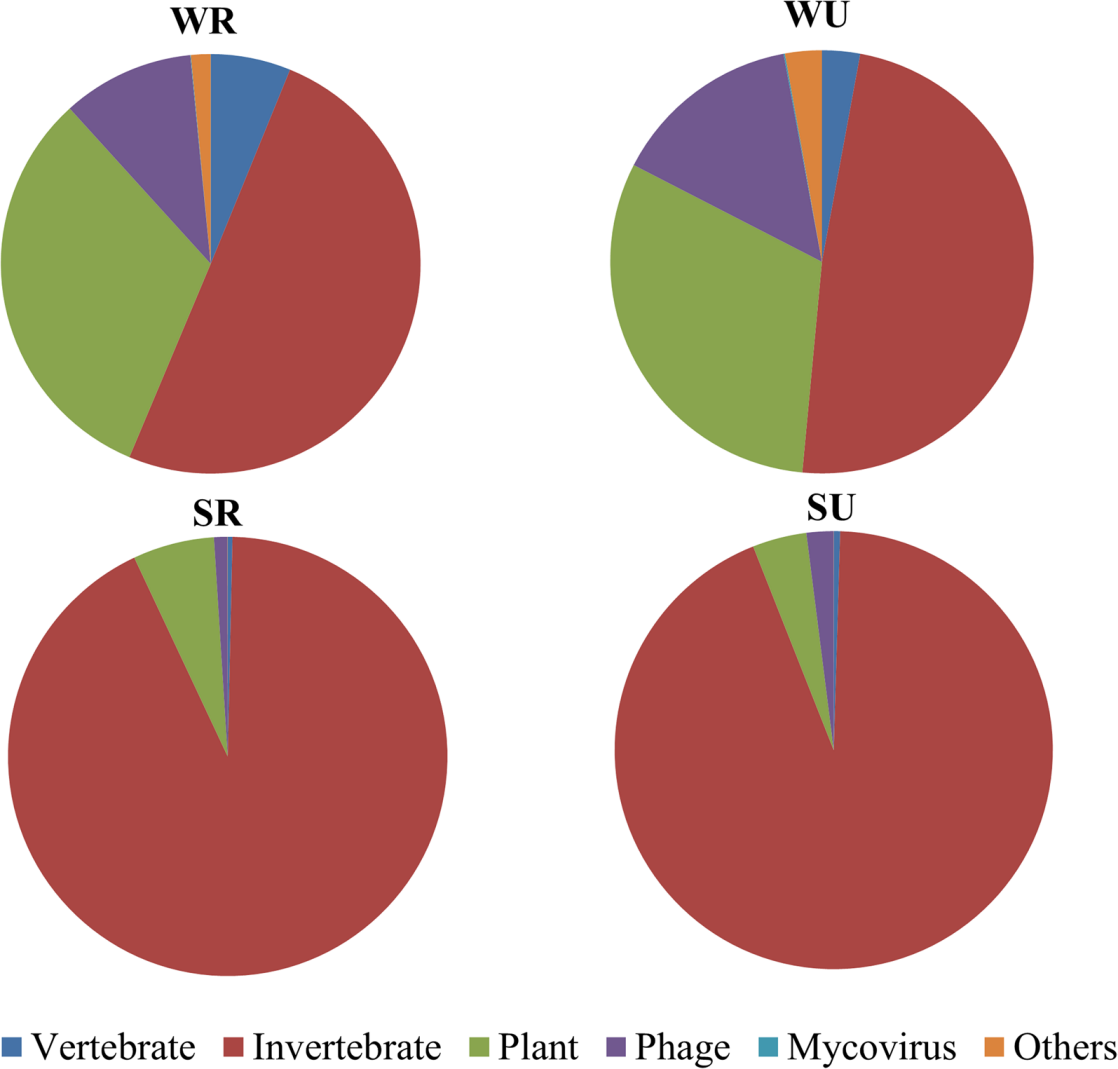
Viral sequences classified by host type. SR: mosquitoes captured in rural areas during summer and autumn; WR: mosquitoes captured in rural areas during winter and spring; SU: mosquitoes captured in urban areas during summer and autumn; WU: mosquitoes captured in urban areas during winter and spring

The viruses detected in *Ae. albopictus* were specific to vertebrates, invertebrates, plants, fungi, bacteria, and protozoa (Fig. 3). Invertebrate viruses were detected with the highest relative abundance in all groups, especially in the mosquitoes that were captured in summer and autumn. Plant viruses and phage also had high relative abundance in all groups, followed by vertebrate viruses. The relative abundance of vertebrate viruses was lower in the mosquitoes captured in summer and autumn than that of the mosquitoes captured in winter and spring.

### Viral composition in *Ae. albopictus*

**Fig. 4:**
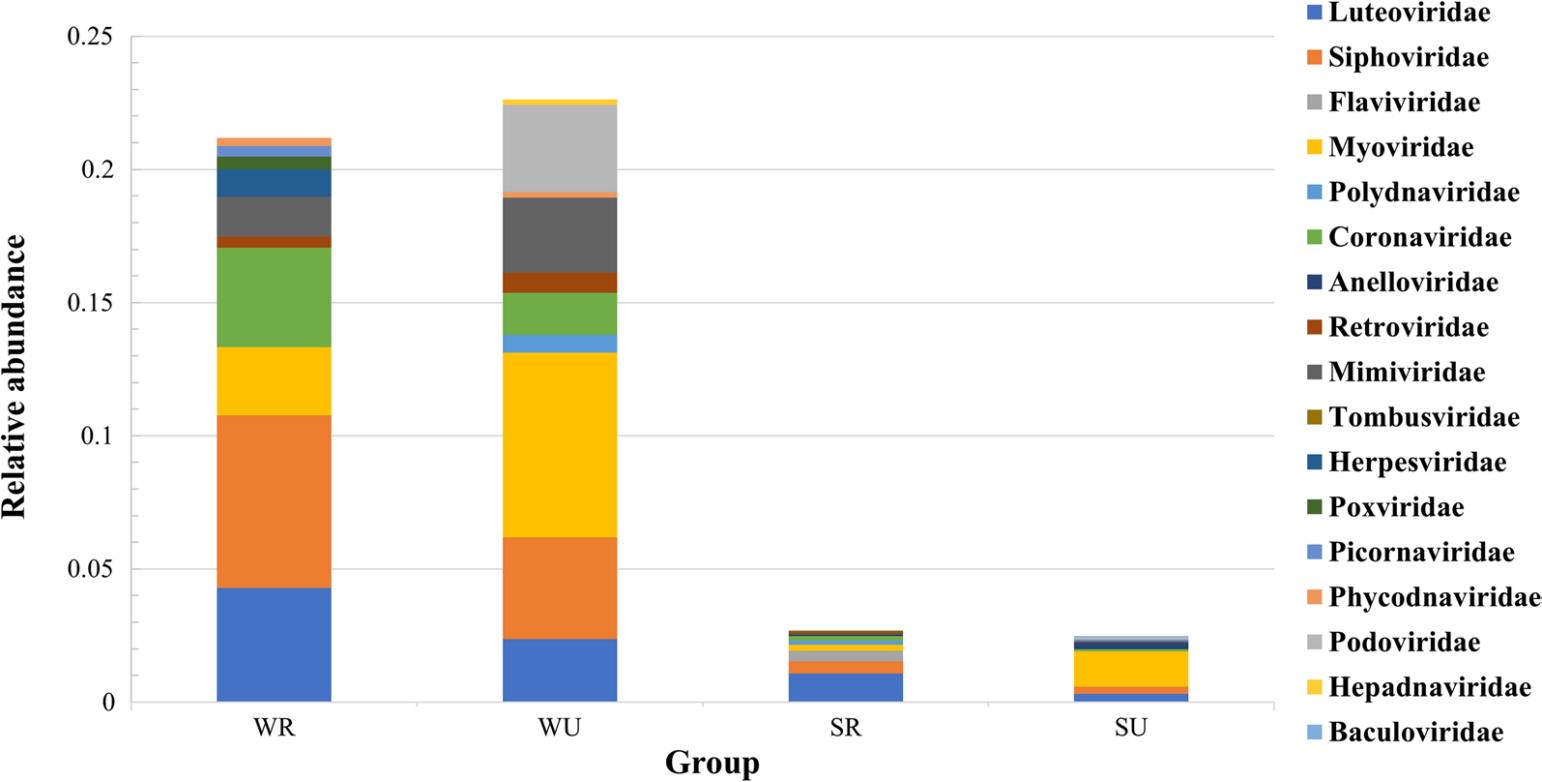
The top ten viral families in the different groups of *Ae. albopictus*. SR: mosquitoes captured in rural areas during summer and autumn; WR: mosquitoes captured in rural areas during winter and spring; SU: mosquitoes captured in urban areas during summer and autumn; WU: mosquitoes captured in urban areas during winter and spring

In rural areas, *Siphoviridae* (6.5%), *Luteoviridae* (4.3%), and *Coronaviridae* (3.7%) were the top three viral families harbored by the mosquitoes captured in winter and spring, while *Luteoviridae* (1.17%), *Siphoviridae* (0.4%), and *Flaviviridae* (0.4%) were the most common viral families harbored by the mosquitoes captured in summer and autumn (Fig. 4). In urban areas, *Myoviridae* was the most common viral family in the mosquitoes captured during all seasons (winter and spring: 7.0%; summer and autumn: 1.3%). *Siphoviridae* (3.8%) and *Podoviridae* (3.3%) were the second and third most common viral families in mosquitoes that were captured during winter and spring, while *Luteoviridae* (0.3%) and *Siphoviridae* (0.3%) were common in mosquitoes captured in summer and autumn. Among the mosquitoes captured in winter and spring, *Luteoviridae* (4.3%), *Siphoviridae* (6.5%), *Coronaviridae* (3.7%), *Herpesviridae* (1.0%), *Poxviridae* (0.5%), and *Picornaviridae* (0.4%) were found with higher relative abundance in mosquitoes captured in rural areas, while *Myoviridae* (7.0%), *Podoviridae* (3.3%), and *Mimiviridae* (2.8%) were more common in mosquitoes captured in urban areas. Higher relative abundance of *Flaviviridae* (0.4%) and *Polydnaviridae* (0.2%) was found in mosquitoes captured in rural areas during summer and autumn, while *Myoviridae* (1.3%), *Anelloviridae* (0.2%), *Picornaviridae* (0.1%), and *Podoviridae* (0.1%) were more common in mosquitoes captured in urban areas during the same seasons.

**Fig. 5:**
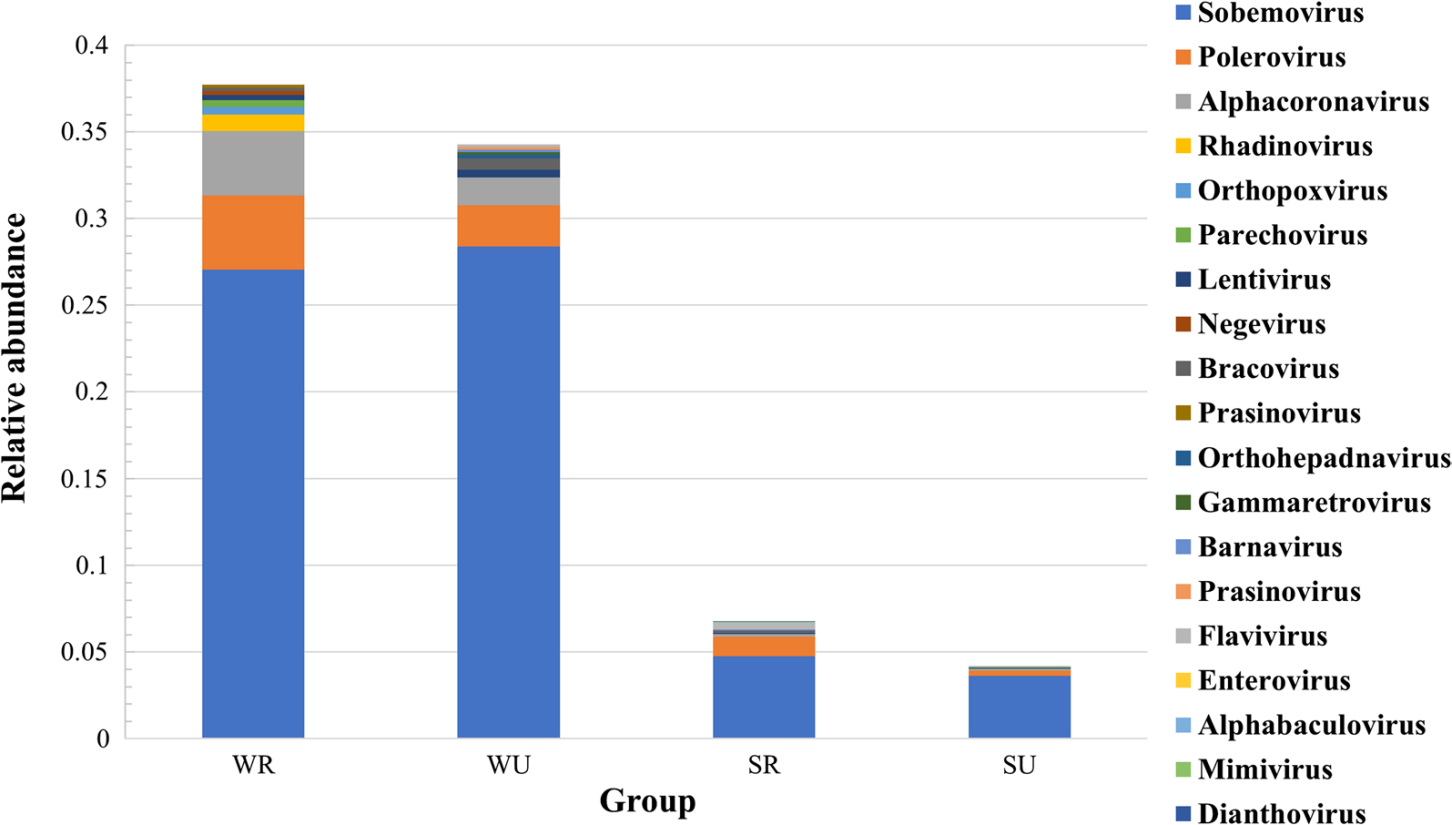
The top ten viral genera in the different groups of *Ae. albopictus*. SR: mosquitoes captured in rural areas during summer and autumn; WR: mosquitoes captured in rural areas during winter and spring; SU: mosquitoes captured in urban areas during summer and autumn; WU: mosquitoes captured in urban areas during winter and spring

At the genus level, *Sobemovirus* and *Polerovirus* were the top two genera in all of the mosquitoes (Fig. 5). *Alphacoronavirus* ranked third in mosquitoes captured in rural areas during winter and spring, *Flavivirus* ranked third in mosquitoes captured in rural areas during summer and autumn, and *Alphacoronavirus* was the third most common viral genus in mosquitoes captured in urban areas during all seasons. In winter and spring, a higher relative abundance of *Rhadinovirus* (0.9%) was detected in mosquitoes captured in rural areas as compared to mosquitoes captured in urban areas. However, in summer and autumn, *Parechovirus* (0.1%) was more common in mosquitoes from urban areas, while a higher relative abundance of *Sobemovirus* (4.8%), *Polerovirus* (1.1%), *Flavivirus* (0.4%), and *Bracovirus* (0.2%) was found in mosquitoes captured in rural areas.

**Fig. 6:**
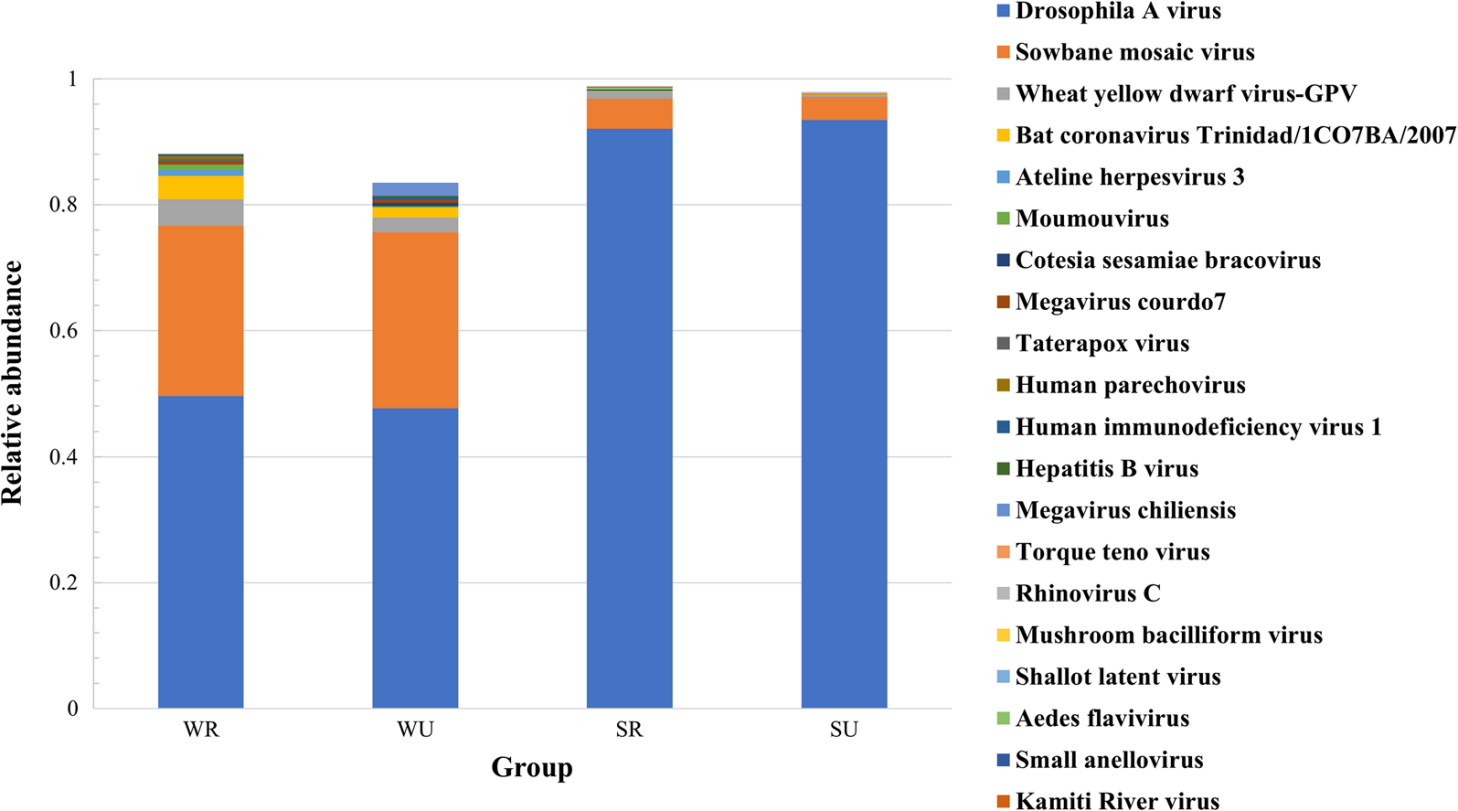
The top ten viral species in the different groups of *Ae. albopictus*. SR: mosquitoes captured in rural areas during summer and autumn; WR: mosquitoes captured in rural areas during winter and spring; SU: mosquitoes captured in urban areas during summer and autumn; WU: mosquitoes captured in urban areas during winter and spring

At the species level, *Drosophila A virus*, *Sowbane mosaic virus*, and *Wheat yellow dwarf virus-GPV* were the top three viral species in all of the mosquitoes (Fig. 6). In both urban and rural areas, *Sowbane mosaic virus*, *Wheat yellow dwarf virus-GPV*, *Bat coronavirus Trinidad/1CO7BA/2007*, *Moumouvirus*, *Megavirus courdo7*, *Human immunodeficiency virus 1*, and *Hepatitis B virus* were found with higher relative abundance in mosquitoes captured during winter and spring, while *Drosophila A virus* and *Mushroom bacilliform virus* were more common in mosquitoes captured during summer and autumn. Mosquitoes trapped in winter and spring from rural areas exhibited higher relative abundance of *Bat coronavirus Trinidad/1CO7BA/2007* (3.7%) and *Ateline herpesvirus 3* (0.9%), while *Hepatitis B virus* (0.2%) and *Megavirus chiliensis* (2.1%) were more common in mosquitoes from urban areas. In summer and autumn, *Torque teno virus* (0.2%) and *Rhinovirus C* (0.1%) exhibited higher relative abundance in mosquitoes from urban areas, while *Aedes flavivirus* (0.4%), *Small anellovirus* (0.1%), and *Kamiti River virus* (0.1%) exhibited higher relative abundance in mosquitoes from rural areas.

### PCR confirmatory testing and phylogenetic analysis

Although *Ae. albopictus* is one of the dominant vectors of ZIKV, DENV, and CHIKV, none of the *Ae. albopictus* in our study was positive for these viruses. Some vertebrate viruses in mosquitoes were detected using PCR, including HPeV and HBV, while negative results were found in the detection of coronavirus, herpesvirus, and TTV (Table 2).

**Table 2:** Viruses detected in *Aedes albopictus*

Virus		Xiongwei village	Nanfang village	Yuexiu district	Tianhe district	Total
Human parechovirus	Female	0 (0/17)	9.1 (2/22)	0 (0/7)	0 (0/18)	3.1 (2/64)
	Male	11.5 (3/26)	2.9 (1/35)	2.9 (1/34)	5.4 (2/37)	5.3 (7/132)
	Total	7.0 (3/43)	5.3 (3/57)	2.4 (1/41)	3.6 (2/55)	4.6 (9/196)
Hepatitis B virus	Female	0 (0/17)	0 (0/22)	0 (0/7)	5.6 (1/18)	1.6 (1/64)
	Male	0 (0/26)	0 (0/35)	0 (0/34)	0 (0/37)	0 (0/132)
	Total	0 (0/43)	0 (0/57)	0 (0/41)	1.8 (1/55)	0.5 (1/196)

**Fig. 7:**
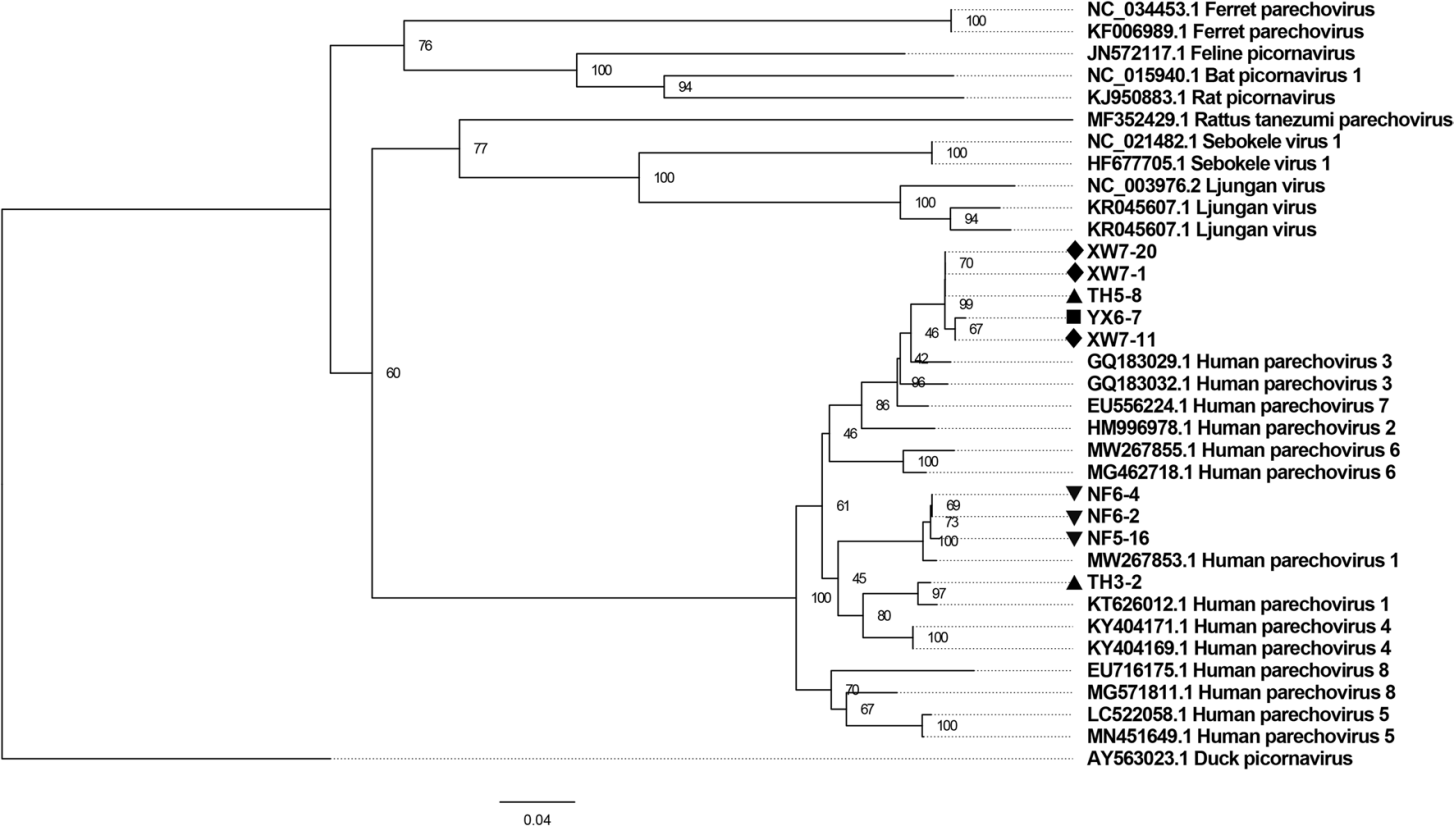
The construction of a phylogenetic tree was based on the screening nucleotide sequence of HPeV from *Ae. albopictus* (MrBayes, GTR + G + I nucleotide substitution model). Twenty-five sequences belonging to different species within genus *Parechovirus* are included for comparison. One sequence belonging to genus *Sapelovirus* is set as outgroup. Percentages of the posterior probability (PP) values are indicated. ▲ Sequences detected in mosquitoes trapped in Tianhe district; ■ Sequence detected in mosquitoes trapped in Yuexiu district; ♦ Sequences detected in mosquitoes trapped in Xiongwei village; ▼ Sequences detected in mosquitoes trapped in Nanfang village

HPeV was detected in seven male and two female mosquito pools. Five of the screening sequences showed a high level of similarity with HPeV 3 sequences, while four sequences were clustered with HPeV 1 (GenBank accession numbers: MW455086-MW455091 and MZ502310- MZ502312) (Fig. 7).

**Fig. 8:**
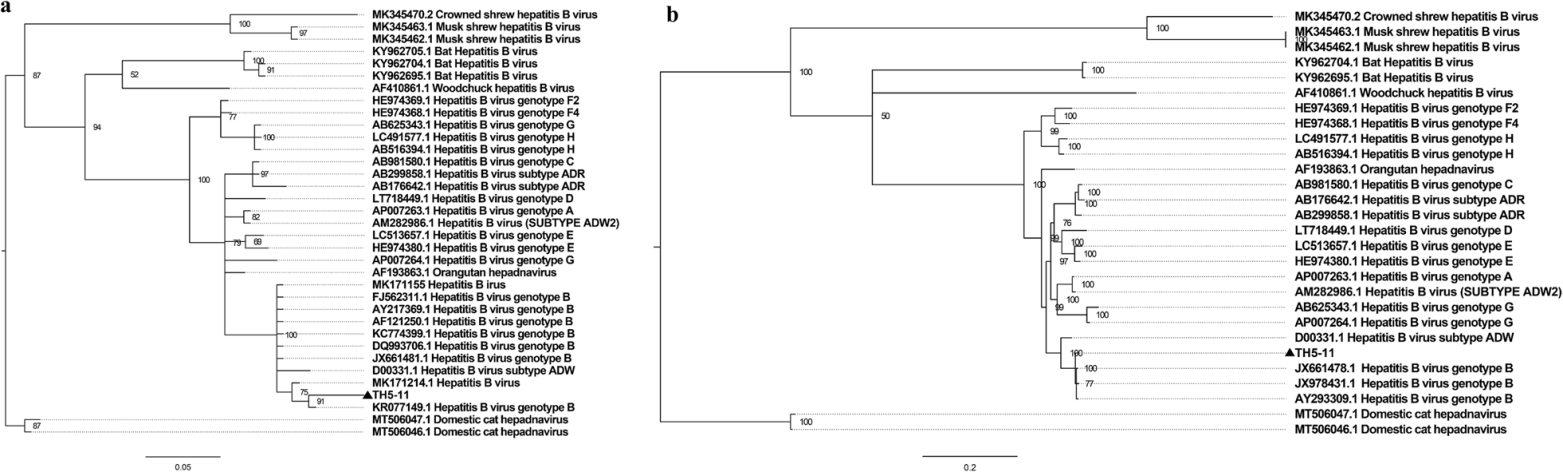
**a** The construction of a phylogenetic tree was based on the screening nucleotide sequence of HBV from *Ae. albopictus* (MrBayes, GTR + G + I nucleotide substitution model). Thirty-two sequences belonging to species *Hepatitis B virus* are included for comparison. Two sequences belonging to species *Domestic cat hepatitis B virus* are set as outgroup. Percentages of the posterior probability (PP) values are indicated. **b** The construction of a phylogenetic tree was based on the near full-length nucleotide sequence of HBV from *Ae. albopictus* (MrBayes, GTR + G + I nucleotide substitution model). Twenty-five sequences belonging to species *Hepatitis B virus* are included for comparison. Two sequences belonging to species *Domestic cat hepatitis B virus* are set as outgroup. Percentages of the posterior probability (PP) values are indicated. ▲ Sequences detected in mosquitoes trapped in Tianhe district

HBV was only detected in one female mosquito pool from an urban area, and a near full-length HBV genome was amplified (TH5-11, GenBank accession number: MW411446). Both the screening sequence and the near full-length genome of HBV were clustered with human HBV genotype B sequences (Fig. 8a and b). The nearly full-length genome exhibited a high level of similarity with the human HBV genotype B sequence (JX661478.1, 99.1%) (Additional file 2: Table S4, Fig. 8b).

## Discussion

Mosquito-borne diseases have greatly influenced human health. *Aedes albopictus* is an invasive animal species, and it is an important reservoir for many arboviruses, including DENV, ZIKV, and CHIKV [5]. Outbreaks of mosquito-borne infectious diseases related to *Ae. albopictus* have been reported. Because of the active movement of humans and climate change, *Ae. albopictus* is found in nearly one third of the regions in China [18]*.* Some mosquito-borne diseases associated with *Ae. albopictus*, such as dengue fever, are prevalent in China [59], and therefore, understanding the virome in *Ae. albopictus* is very important for the prevention and control of mosquito-borne diseases. Compared with traditional methods, viral metagenomics is more efficient for viral identification and discovery [60]. Many studies have used viral metagenomics to investigate the viruses in mosquitoes [39, 61, 62]. However, few studies have focused on *Ae. albopictus*.

In this study, viral metagenomics was performed to investigate the virome in adult *Ae. albopictus* captured in different areas and during different seasons in Guangzhou City, Guangdong Province, China. We also investigated the prevalence and genetic diversity of several arboviruses and vertebrate viruses in *Ae. albopictus* using PCR. To the best of our knowledge, this is the first study to reveal and compare the viral composition in adult *Ae. albopictus* captured in different areas and during different seasons in Guangzhou, China.

The viral composition in *Ae. albopictus* varied mainly between seasons, and the viral composition between mosquitoes captured in different areas during the same season showed a high level of similarity (Figs. 1 and 2). However, a previous study revealed a significant difference in the viral communities when comparing mosquitoes captured in different regions [61]. The high level of similarity in viral composition between mosquitoes captured in rural and urban areas in our study might be explained by the following: first, we collected the mosquitoes in urban and rural areas in the same city, and the sampling areas are close, with a maximum straight-line distance between Xiongwei village and Keyuan community of 47 km; second, the large population and active movement of humans in Guangzhou facilitated the spread of *Ae. albopictus* [30].

Large amounts of the sequences detected in the mosquitoes from our study cannot be annotated to known viral species, and this was consistent with the results of a previous study [63]. It seems that *Ae. albopictus* harbors a large number of novel viruses, and further studies are required to investigate these unknown viruses*.* Even though there was only a small proportion of detected sequences that could be annotated as known viruses, more than 50 viral families were annotated in adult *Ae. albopictus*, and these viruses were specific to vertebrates, invertebrates, plants, fungi, bacteria, and protozoa. Invertebrate viruses appeared with the highest relative abundance in all samples of *Ae. albopictus*, which is consistent with the results for *Culex* mosquitoes [39]. Large numbers of bacteriophages were detected, including members in *Myoviridae*, *Siphoviridae*, and *Podoviridae* [64]. Plant viruses were also found, such as members in *Luteoviridae* [65]. Some viruses that can cause diseases in humans and animals were detected, indicating the wide range of blood hosts for *Ae. albopictus* in Guangzhou.

Low temperatures limit insect development and activity, as well as the replication of viruses in insects [66]. Interestingly, the number of viral species in *Ae. albopictus* captured in winter and spring was slightly greater than that in *Ae. albopictus* captured in summer and autumn. In addition, a lower relative abundance of vertebrate viruses was found in *Ae. albopictus* captured in summer and autumn as compared to that in *Ae. albopictus* captured in winter and spring. This might be associated with the average winter temperature in Guangzhou (15.9 °C) [67], which is high enough that *Ae. albopictus* is active all year round in this city. A previous study showed that *Ae. albopictus* can still feed on humans and animals, and produce eggs in early winter in Guangzhou [68]. In addition, the sex ratio of emerged adults in early winter, such as in November (female/male = 1.2), is higher than that in some summer months (female/male = 0.71 in August), indicating that there may be a larger proportion of female *Ae. albopictus* in winter than in summer [68].

PCR or viral metagenomic analysis indicated that none of the samples was positive for ZIKV, DENV, or CHIKV. This is consistent with the prevalence of the relative diseases in human populations: none of the related mosquito-borne diseases were reported in the sampling areas at the time the mosquitoes were captured. In the future, studies should be continued to survey the virome in *Ae. albopictus* in order to monitor and prevent related mosquito-borne diseases.

Large numbers of vertebrate viruses were detected in our study. Members within *Coronaviridae* can cause human diseases [69]. Using viral metagenomics, *Coronaviridae* was found in *Ae. albopictus*, and it was the most abundant vertebrate viral family in our study. However, none of the samples was positive for it using PCR. This discrepancy might be explained by the high sensitivity of viral metagenomics. A higher relative abundance of *Coronaviridae* was found in mosquitoes captured during winter and spring. Interestingly, the circulation of coronaviruses in human and animal populations in Guangzhou City also exhibited a peak of coronavirus infection in winter and spring [70]. It seems that xenosurveillance is feasible, and the relative abundance of *Coronaviridae* in *Ae. albopictus* can provide some information regarding the disease prevalence in human populations. *Bat coronavirus Trinidad/1CO7BA/2007* was the only species detected within *Coronaviridae.* This virus was first detected in bats in America [71]. Its detection in *Ae. albopictus* indicated that this virus had already spread to the bats in Guangzhou, China. Some pathogenetic coronaviruses might have originated from bats [72], and therefore, studies on these animals is necessary to prevent the transmission of emerging viruses.

A previous study reported the detection of *Herpesviridae* in *Culex* mosquitoes [61]. In our study, *Herpesviridae* was the second most abundant vertebrate viral family in *Ae. albopictus*. Different species of herpesviruses were detected, including *Ateline herpesvirus 3*, *Macacine herpesvirus 1*, *Caviid herpesvirus 2*, *Gallid herpesvirus 1*, *Human herpesvirus 6*, and *Human herpesvirus 6B*, suggesting that many animals in Guangzhou were infected by herpesviruses. Like *Coronaviridae*, a higher relative abundance of *Herpesviridae* was found in *Ae. albopictus* captured in winter and spring than that in *Ae. albopictus* captured in summer and autumn, which might be explained by the seasonal dynamics of herpesviruses in human and animal populations [73].

Sequences annotated as *Anelloviridae* have been detected in other species of mosquitoes, such as *Anopheles* mosquitoes [62]. Three members of *Anelloviridae* were annotated in our study, including *Torque teno virus*, *Small anellovirus*, and *Torque teno sus virus 1a*, indicating that humans and pigs in Guangzhou were infected by anelloviruses. However, PCR results indicated that none of the mosquito pools was positive for TTV, which might also be explained by the high sensitivity of viral metagenomics. The pathogenicity of TTV is still unknown, but it is prevalent in humans [74], and mother-to-infant vertical transmission of this virus is known to occur [75]. Studies should be performed to investigate the relationship between TTV and human diseases. Pigs are often co-infected with torque teno sus virus and other viruses, especially the porcine circovirus, and it may pose a potential threat to swine herds [39].

Like *Coronaviridae* and *Herpesviridae*, a higher relative abundance of *Picornaviridae* was also found in mosquitoes captured in winter and spring. A similar seasonal pattern for picornaviruses was found in some blood hosts of *Ae. albopictus* [76]. Three viral species within *Picornaviridae* were detected, including *Human parechovirus*, *Rhinovirus C*, and *Oscivirus A*. HPeV and rhinovirus C are human pathogenic viruses related to gastrointestinal diseases and respiratory diseases, respectively [77]. It seems that humans in Guangzhou were infected by parechovirus and rhinovirus, and it would be a worthy endeavor to further study these viruses for disease prevention and control. Osciviruses originate from amphibians and birds [78, 79], suggesting that *Ae. albopictus* also feeds on these animals. The PCR results indicated that nine pooled samples were positive for HPeV. The HPeV sequences obtained in our study were clustered with HPeV 1 and 3 sequences. HPeV 1 can cause acute gastroenteritis, and it is the most frequently identified member within the genus *Parechovirus* [80–82]. Disease outbreak related to HPeV 3 has been reported in humans [83]. To prevent the relative diseases in Guangzhou, mosquito surveillance is beneficial because it can provide rapid identification of the spread of emerging HPeV types. In our study, most of the HPeV sequences were detected in male mosquitoes. Male *Ae. albopictus* do not need a blood meal, and it was unlikely that they acquired this virus from humans by mosquito bite. Male *Ae. albopictus* might have acquired HPeV by vertical transmission or from the environment, such as from raw sewage [84]. However, there is no evidence indicating that HPeV can be transmitted vertically. Thus, it is likely that the HPeV in male *Ae. albopictus* was obtained from the environment. Surveillance of the virome in mosquitoes not only provides data that reflects the viral infection status in humans and animals, but also provides information regarding the viruses in the environment.

*Hepatitis B virus* was the only viral species within *Hepadnaviridae* detected by viral metagenomics. A higher relative abundance of *Hepatitis B virus* was found in mosquitoes captured during winter and spring than that in mosquitoes captured during summer and autumn, which may be explained by the seasonal fluctuations in HBV DNA levels in humans [85]. PCR results indicated that one female mosquito pool collected in an urban area was positive for HBV, and the first near full-length genome of HBV (TH5-11) from mosquitoes was amplified. The screening sequence and the near full-length genome showed a high level of similarity with human HBV genotype B sequences. A previous study reported that HBV genotype B is common in Asia [86], and the results in our study similarly indicated that HBV genotype B is prevalent in humans in Guangzhou. Mosquito bites may transmit HBV to laboratory animals [87]. However, up until now, there is no evidence showing that mosquitoes can transmit HBV to humans. Even though we amplified the near full-length genome of HBV in the current study, the most likely explanation for this detection is that the mosquitoes fed on HBV-infected humans. Experiments are needed to determine the role of *Ae. albopictus* in HBV transmission.

This research can further our understanding of the virome in adult *Ae*. *albopictus* in Guangzhou City. We also demonstrated that there is a wide circulation of diverse vertebrate viruses in *Ae*. *albopictus*. In addition, the relative abundance of vertebrate viruses in *Ae*. *albopictus* was in accordance with the disease prevalence in humans and animals, suggesting that surveillance of the virome in adult *Ae*. *albopictus* not only provides information to prevent mosquito-borne diseases but also forms a framework for the surveillance, prevention, and control of other human diseases. Surveillance of the virome in *Ae*. *albopictus* can also provide information regarding the viruses in the environment.

There are some limitations to our study. First, we did not pool the mosquitoes by sex in our viral metagenomic studies, which might affect the viral community composition within them. Second, midgut and salivary gland dissections were not performed to identify where the viruses were located. Third, the head, legs, and wings of the mosquitoes were not removed in our study. In the future, mosquito heads, legs, and wings should be removed to prevent PCR inhibition and reduce the host genome. Our findings should be confirmed by more rigorous studies with larger sample sizes.

## Conclusions

This study revealed the viral composition of adult *Ae*. *albopictus* captured in different areas and during different seasons in Guangzhou City. The viral composition in *Ae*. *albopictus* varied mainly between seasons. A higher relative abundance of some vertebrate viruses was found in the mosquitoes trapped in winter and spring, which was consistent with the seasonal patterns of the related viruses in humans and animals. HPeV and HBV were detected in *Ae*. *albopictus* using PCR. Although the first near full-length genome of HBV from *Ae*. *albopictus* was amplified, additional research is still needed to decipher whether *Ae*. *albopictus* plays a role in the transmission of HBV. In the future, surveillance of the virome in *Ae. albopictus* should be continued to provide information for the prevention and control of mosquito-borne diseases. In addition, xenosurveillance is feasible, and the surveillance of the virome in *Ae*. *albopictus* can also form a framework for surveillance, prevention, and control of other human diseases*.*

## Supplementary Information

**Additional file 1**: **Table S1**. Mosquitoes pooled for viral metagenomic analysis. **Table S2**. Mosquito pools for viral detection using PCR. **Table S3**. Sequencing results of viral metagenomics

**Additional file 2**: **Table S4**. Nucleotide sequence identity for the near full-length genomic sequences of hepatitis B virus (HBV) from *Aedes albopictus*, humans, bats and woodchuck.

## Data Availability

All of the data generated or analyzed during this study are included in this manuscript and the supplementary information files. Sequences of HBV and HPeV were uploaded to the NCBI database (GenBank accession numbers: MW455086-MW455091, MZ502310-MZ502312 and MW411446). The viral metagenomic data were uploaded to the NCBI Sequence Read Archive (SRA) repository (SRA number: SRP304029).

## Abbreviations

*Ae. albopictus*: *Aedes albopictus*
CHIKV: Chikungunya virus
DENV: Dengue virus
HBV: Hepatitis B virus
HPeV: Human parechovirus
NCBI NT database: National Center for Biotechnology Information nucleotide database
NCBI NR database: National Center for Biotechnology Information non-redundant protein sequence database
ORFs: Open reading frames
PBS: Phosphate-buffered saline
PCA: Principal component analysis
PCoA: Principal coordinates analysis
PP: Posterior probability
TTV: Torque teno virus
YFV: Yellow fever virus
ZIKV: Zika virus
