# Supplementary material for: Virome in adult Aedes albopictus captured during different seasons in Guangzhou City, China
Source: Parasit Vectors. 2021 Aug 18;14:415. doi: 10.1186/s13071-021-04922-z (PMC8371599; doi:10.1186/s13071-021-04922-z)
Supplement: Supplementary file 1 — Additional file 1: Table S1. Mosquitoes pooled for viral metagenomic analysis. Table S2. Mosquito pools for viral detection using PCR. Table S3. Sequencing results of viral metagenomics [file 13071_2021_4922_MOESM1_ESM.docx]

**Additional file 1: Table S1.** Mosquitoes pooled for viral metagenomic analysis.

| Sample name | Mosquito group ^a^ | Species | Date | No. of mosquitoes |
| --- | --- | --- | --- | --- |
| XW-2017-09 | SR | *Ae. albopictus* | 2017-09 | 20 |
| XW-2017-10 | SR | *Ae. albopictus* | 2017-10 | 33 |
| XW-2017-11 | WR | *Ae. albopictus* | 2017-11 | 50 |
| XW-2017-12 | WR | *Ae. albopictus* | 2017-12 | 0 |
| XW-2018-01 | WR | *Ae. albopictus* | 2018-01 | 2 |
| XW-2018-02 | WR | *Ae. albopictus* | 2018-02 | 5 |
| XW-2018-03 | WR | *Ae. albopictus* | 2018-03 | 21 |
| XW-2018-04 | - | *Ae. albopictus* | 2018-04 | - |
| XW-2018-05 | - | *Ae. albopictus* | 2018-05 | - |
| XW-2018-06 | SR | *Ae. albopictus* | 2018-06 | 22 |
| XW-2018-07 | SR | *Ae. albopictus* | 2018-07 | 32 |
| XW-2018-08 | SR | *Ae. albopictus* | 2018-08 | 28 |
| NF-2017-09 | SR | *Ae. albopictus* | 2017-09 | 30 |
| NF-2017-10 | SR | *Ae. albopictus* | 2017-10 | 30 |
| NF-2017-11 | WR | *Ae. albopictus* | 2017-11 | 64 |
| NF-2017-12 | WR | *Ae. albopictus* | 2017-12 | 8 |
| NF-2018-01 | WR | *Ae. albopictus* | 2018-01 | 1 |
| NF-2018-02 | WR | *Ae. albopictus* | 2018-02 | 22 |
| NF-2018-03 | WR | *Ae. albopictus* | 2018-03 | 32 |
| NF-2018-04 | - | *Ae. albopictus* | 2018-04 | - |
| NF-2018-05 | - | *Ae. albopictus* | 2018-05 | - |
| NF-2018-06 | SR | *Ae. albopictus* | 2018-06 | 27 |
| NF-2018-07 | SR | *Ae. albopictus* | 2018-07 | 29 |
| NF-2018-08 | SR | *Ae. albopictus* | 2018-08 | 31 |
| YX-2017-09 | SU | *Ae. albopictus* | 2017-09 | 20 |
| YX -2017-10 | SU | *Ae. albopictus* | 2017-10 | 32 |
| YX -2017-11 | WU | *Ae. albopictus* | 2017-11 | 30 |
| YX -2017-12 | WU | *Ae. albopictus* | 2017-12 | 41 |
| YX -2018-01 | WU | *Ae. albopictus* | 2018-01 | 0 |
| YX -2018-02 | WU | *Ae. albopictus* | 2018-02 | 17 |
| YX -2018-03 | WU | *Ae. albopictus* | 2018-03 | 16 |
| YX -2018-04 | - | *Ae. albopictus* | 2018-04 | - |
| YX -2018-05 | - | *Ae. albopictus* | 2018-05 | - |
| YX -2018-06 | SU | *Ae. albopictus* | 2018-06 | 20 |
| YX -2018-07 | SU | *Ae. albopictus* | 2018-07 | 30 |
| YX -2018-08 | SU | *Ae. albopictus* | 2018-08 | 49 |
| TH-2017-09 | SU | *Ae. albopictus* | 2017-09 | 20 |
| TH -2017-10 | SU | *Ae. albopictus* | 2017-10 | 31 |
| TH -2017-11 | WU | *Ae. albopictus* | 2017-11 | 20 |
| TH -2017-12 | WU | *Ae. albopictus* | 2017-12 | 45 |
| TH -2018-01 | WU | *Ae. albopictus* | 2018-01 | 0 |
| TH -2018-02 | WU | *Ae. albopictus* | 2018-02 | 20 |
| TH -2018-03 | WU | *Ae. albopictus* | 2018-03 | 19 |
| TH -2018-04 | - | *Ae. albopictus* | 2018-04 | - |
| TH -2018-05 | - | *Ae. albopictus* | 2018-05 | - |
| TH -2018-06 | SU | *Ae. albopictus* | 2018-06 | 21 |
| TH -2018-07 | SU | *Ae. albopictus* | 2018-07 | 41 |
| TH -2018-08 | SU | *Ae. albopictus* | 2018-08 | 31 |

^a^ SR: Mosquitoes captured in rural areas during summer and autumn. WR: Mosquitoes captured in rural areas during winter and spring. SU: Mosquitoes captured in urban areas during summer and autumn. WU: Mosquitoes captured in urban areas during winter and spring.

**Additional file 1: Table S2.** Mosquito pools for viral detection using PCR.

| Season | Location | Mosquito sex | No. of mosquitoes | No. of pools |
| --- | --- | --- | --- | --- |
| Winter and spring | XW | Female | 110 | 17 |
|  | NF | Female | 161 | 22 |
|  | YX | Female | 38 | 7 |
|  | TH | Female | 112 | 18 |
|  | Subtotal | Female | 421 | 64 |
| Summer and autumn | XW | Male | 384 | 26 |
|  | NF | Male | 521 | 35 |
|  | YX | Male | 490 | 34 |
|  | TH | Male | 540 | 37 |
|  | Subtotal | Male | 1935 | 132 |
|  | Total |  | 2356 | 196 |

XW: Xiongwei village; NF: Nanfang village; YX: Yuexiu district; TH: Tianhe district

**Additional file 1: Table S3.** Sequencing results of viral metagenomics.

| Mosquito group ^a^ | No. of mosquitoes | No. of raw reads | No. of clean reads | No. of contigs | No. of ORFs |
| --- | --- | --- | --- | --- | --- |
| SR | 282 | 46,603,020 | 26,719,846 | 59,259 | 28,148 |
| SU | 295 | 43,653,684 | 28,612,447 | 43,478 | 21,000 |
| WR | 205 | 44,596,328 | 30,069,990 | 37,006 | 16,981 |
| WU | 208 | 45,197,544 | 24,453,828 | 48,265 | 22,989 |

^a^ SR: Mosquitoes captured in rural areas during summer and autumn. WR: Mosquitoes captured in rural areas during winter and spring. SU: Mosquitoes captured in urban areas during summer and autumn. WU: Mosquitoes captured in urban areas during winter and spring.
