# Supplementary material for: Virome in adult Aedes albopictus captured during different seasons in Guangzhou City, China
Source: Parasit Vectors. 2021 Aug 18;14:415. doi: 10.1186/s13071-021-04922-z (PMC8371599; doi:10.1186/s13071-021-04922-z)
Supplement: Supplementary file 2 — Additional file 2: Table S4. Nucleotide sequence identity for the near full-length genomic sequences of hepatitis B virus (HBV) from Aedes albopictus, humans, bats and woodchuck. [file 13071_2021_4922_MOESM2_ESM.docx]

**Additional file 2: Table S4.** Nucleotide sequence identity for the near full-length genomic sequences of hepatitis B Virus (HBV) from *Aedes albopictus*, humans, bats and woodchuck

|  | TH5-11 | AP007263.1 HBV genotype A | JX661478.1 HBV genotype B | JX978431.1 HBV genotype B | AY293309.1 HBV genotype B | AB981580.1 HBV genotype C | LT718449.1 HBV genotype D | KY962704.1 Bat hepatitis B virus | KY962695.1 Bat hepatitis B virus | AF410861.1 Woodchuck hepatitis B virus |
| --- | --- | --- | --- | --- | --- | --- | --- | --- | --- | --- |
| TH5-11 | ID | 0.517 | 0.991 | 0.989 | 0.885 | 0.509 | 0.506 | 0.363 | 0.365 | 0.462 |
| AP007263.1 HBV genotype A | 0.517 | ID | 0.517 | 0.516 | 0.421 | 0.918 | 0.919 | 0.646 | 0.649 | 0.394 |
| JX661478.1 HBV genotype B | 0.991 | 0.517 | ID | 0.991 | 0.888 | 0.509 | 0.506 | 0.364 | 0.365 | 0.461 |
| JX978431.1 HBV genotype B | 0.989 | 0.516 | 0.991 | ID | 0.887 | 0.506 | 0.506 | 0.363 | 0.365 | 0.461 |
| AY293309.1 HBV genotype B | 0.885 | 0.421 | 0.888 | 0.887 | ID | 0.412 | 0.408 | 0.299 | 0.3 | 0.413 |
| AB981580.1 HBV genotype C | 0.509 | 0.918 | 0.509 | 0.506 | 0.412 | ID | 0.905 | 0.643 | 0.643 | 0.396 |
| LT718449.1 HBV genotype D | 0.506 | 0.919 | 0.506 | 0.506 | 0.408 | 0.905 | ID | 0.64 | 0.644 | 0.393 |
| KY962704.1 Bat hepatitis B virus | 0.363 | 0.646 | 0.364 | 0.363 | 0.299 | 0.643 | 0.64 | ID | 0.988 | 0.404 |
| KY962695.1 Bat hepatitis B virus | 0.365 | 0.649 | 0.365 | 0.365 | 0.3 | 0.643 | 0.644 | 0.988 | ID | 0.404 |
| AF410861.1 Woodchuck hepatitis B virus | 0.462 | 0.394 | 0.461 | 0.461 | 0.413 | 0.396 | 0.393 | 0.404 | 0.404 | ID |

TH5-11: The near-full length genomic sequence of HBV obtained in this study.
